# DNA Dispose, but Subjects Decide. Learning and the Extended Synthesis

**DOI:** 10.1007/s12304-015-9242-3

**Published:** 2015-05-27

**Authors:** Markus Lindholm

**Affiliations:** Norwegian Institute for Water Research (NIVA), Gaustadalléen 21, 0349 Oslo, Norway; Rudolf Steiner University College, Dahls gate 30, 0260 Oslo, Norway

**Keywords:** Learning, Decision making, Speciation, Darwin’s finches, The modern synthesis, The extended synthesis

## Abstract

Adaptation by means of natural selection depends on the ability of populations to maintain variation in heritable traits. According to the Modern Synthesis this variation is sustained by mutations and genetic drift. Epigenetics, evodevo, niche construction and cultural factors have more recently been shown to contribute to heritable variation, however, leading an increasing number of biologists to call for an extended view of speciation and evolution. An additional common feature across the animal kingdom is learning, defined as the ability to change behavior according to novel experiences or skills. Learning constitutes an additional source for phenotypic variation, and change in behavior may induce long lasting shifts in fitness, and hence favor evolutionary novelties. Based on published studies, I demonstrate how learning about food, mate choice and habitats has contributed substantially to speciation in the canonical story of Darwin’s finches on the Galapagos Islands. Learning cannot be reduced to genetics, because it demands decisions, which requires a subject. Evolutionary novelties may hence emerge both from shifts in allelic frequencies and from shifts in learned, subject driven behavior. The existence of two principally different sources of variation also prevents the Modern Synthesis from self-referring explanations.

## Background

A common feature of living things is the potential disparity between population growth and limited resources. This possible mismatch favors inheritable traits which improve survival and reproductive success, and led Charles Darwin and Alfred Russel Wallace to the answer of the mystery of speciation: adaptation by natural selection. The mechanism potentially affects all fields of life, and constitutes a core principle in evolutionary biology. Natural selection rests on two premises which are, however, less intuitive. Heritable traits must vary, and populations must maintain persistent affiliations to specific environments. Selection counteracts variation, a point that led many biologists to abandon Darwin’s theory a century ago. How variation is maintained despite headwinds from selection was elucidated by the new science of genetics, which identified heritable mutations and genetic drift as molecular sources for variation. Mutation-driven change in allelic frequencies maintains fitness differences, which by means of selection leads to a gradual adaptation of the whole population (Fisher 1930; Dobzhansky 1937; Huxley 1942). The mystery of creation hence seemed largely solved within the framework emerging as the Modern Synthesis in the middle of the 20th century.

The identified sources for heritable variation brought genes to the center of evolutionary thought. During the following decades, it became increasingly common to view life as optimized for genes rather than for organisms. The view of genes as “selfish replicators” and phenotypes as perishable vehicles emerged as compelling in both the academic community and the public throughout the last decades of the century (Hamilton 1971; Dawkins 1976).

### Epigenetics and Evodevo

The Modern Synthesis, however, gradually opened for new questions, as well. One of them addressed the complex assortment of proteins and chemicals which regulate the activation and transcription of genes, assembling to what now is known as the epigenome. Epigenetic regulation controls cell differentiation, maintains the stable phenotype and accommodates the organism to its local environment through histones and methylation of selected DNA sequences. Methylated areas of DNA are commonly reset in the germline, preventing transfer of phenotypic acquired information to descendants. Cases of transgenerational and persistent epigenetic inheritance proliferate, however (Jablonka and Lamb 2005; Smits et al. 2008; Shapiro 2011), thus pointing to selectable traits and Darwinian evolution mediated by extragenomic information. For instance, phenotypic differences between man and chimpanzees are epigenetic rather than genetic, and have been so since the human-chimp lineage split, 6–8 million years ago (Martin et al. 2011). Whether the epigenetic information is inherited along the germline as DNA or along other parts of chromosomes (Boffelli and Martin 2012) remains open.

Moreover, the Modern Synthesis had assumed gradual change of genes that are involved in the evolution of convergent structures. Puzzling similar morphology and behavioral habits in quite different taxa, as the fungi-cultivation of African termites and Nearctic leaf cutting ant or the nearly identical eye morphology across the animal kingdom, were both viewed as adaptive responses to similar environments. The discovery of homologous regulatory genes, however, partly buried deep in phylogeny, has shed new light on the molecular background for such convergences (Gehring 1996; Stern 2013). Gene duplication, gene redundancy and associated buffering, moreover, allow phenotypes to maintain genetic variability, and enable genetic re-activation through alternative developmental pathways, through what is now acknowledged as evolutionary development, or simply evodevo (Rutherford 2000; Müller 2007; Carroll 2008; Badyaev 2009). Hence, diversification of common structures is not solely a matter of Darwinian adaptation, but has been guided and constrained by shifts in signaling pathways during early development (Helms et al. 2005; Ahi et al. 2014), and the genotype/phenotype relationship has clearly emerged as more complex than originally assumed: “Thinking of genomes as blueprints has (…) delayed the incorporation of developmental biology into evolutionary theory, and is still delaying the expansion of the general concept of heritable information” (Pigliucci and Boudry 2011, p. 455).

### Organism-Environment Interactions

Other new questions have emerged from studies of organism-environment interactions. The conventional view considered speciation as a unilateral process caused by environmental factors which run detached from the organism, where genetic change was driven independent from the ecological niche. Indeed, organisms alter their surroundings and thereby affect fitness of other species, and speciation commonly involves elements of co-evolution (Van Valen 1973; Stenseth and Maynard Smith 1984; Lawrence et al. 2012). Charles Darwin recognized the importance of earthworms for soil development and vegetation; Vladimir Vernadsky claimed that atmospheric features were affected by photosynthesis, and James Lovelock and Lynn Margulis introduced the Gaia theory of interconnected organisms (Margulis and Lovelock 1974). Though compelling, such findings do not predate the core message from the Modern Synthesis, as these features still rest on genomic shifts independent from the environmental needs of phenotypes. The organism-environment interactions become more intriguing, however, where species purposely facilitate their environments in order to gain a better fit to their own needs, through mechanisms now recognized as niche construction (Lewontin 2001; Odling-Smee et al. 2003). A familiar example are beaver dams, which improve the environment for their constructors and allow access to new resources and shelter against predators. Beavers modify their environment in order to gain a better match with their inherent needs. Other cases of niche construction are the arboreal night beds of hominid apes, bird nests which allow the offspring to grow up in “artificial” environments, or oaks, which through litter fall facilitate the soil to the needs of their own seeds. Extensive niche construction is also seen in anthills, wasp nests or earthworm burrows. Such modified niches affect fitness and alter the frame premises of Darwinian selection on their constructors.

The most extensive niche constructor is certainly *Homo sapiens*, which stabilizes the local climatic environment through houses and clothing, alters his life history by use of medical treatment and designs his diet by cultivation of fruits, farming, vegetables and cereal corn. Secondary genetic impacts of these niche modifications are well acknowledged (Perry et al. 2007; Tishkoff et al. 2007). The cultural habit of cooking has certainly contributed to the accelerating encephalization in our lineage, as the present human brain is far too energy-consuming to be maintained by a natural diet. Cooking both facilitates chewing and digesting, detoxifies, disinfects and improves uptake of nutrients and energy. The accelerating growth of the hominid brain during the last million years corresponds closely with reduced gastrointestinal tracts, chewing muscles, jaws and teeth (Leonard et al. 2007; Wrangham 2009), and is considered as an adaptation to the cultural habit of a pre-processed diet. The cranial morphology in humans, as well as the narrow hip and reduced abdomen hence reflects adaptations to a self-constructed dietary niche (Wrangham and Conklin-Brittain 2003; Turner and Thompson 2013).

Constructed habitats may be transferred to descendants by means of “ecological inheritance”, as in colonies of birds or rodents. These stabilize behavioral modes and are inherited similarly to farms, generating a “silver spoon” effect in cultural dependent fitness (Avital and Jablonka 2000; van de Pol et al. 2006; East et al. 2009). The gene-environment interdependence established through niche construction creates a complete two-way bridge between organism and its niche, as the modified environment in turn constitutes the niche to which descendants will be adapted to. Seemingly paradoxically, species are to some extent “adapted to themselves” (Turner 2000).

Such additional sources of variation have increasingly complicated the framework of the Modern Synthesis, and has assembled to what now is sometimes called the *extended synthesis* (Kutschera and Niklas 2004; Pigliucci 2007; Pfennig et al. 2010; Pigliucci and Müller 2010; Danchin et al. 2011a; Bonduriansky 2012; Lindholm 2012; Laland et al. 2014). These extensions do not at all preclude the essential position of mutations for evolutionary novelties, but their proponents claim that more can be said about how variation is maintained and why certain mutations are beneficial. The Modern Synthesis clearly identified a central determinant of phenotypic change and evolution. But organisms modify the niches which subsequently favor genetic adaptations. Organisms are therefore themselves potential causes for subsequent genetic change in their own lineage - which lead us to the phenomenon of learning.

### Subjects Learn

Song is the hidden joy of the larch egg....

André Bjerke (Norwegian poet)

Objects and dead things are exposed to external forces in a mechanistic regime of cause and effect. They lack internality. Subjects are different. They are embedded in and surrounded by meaningful signs (King 2004). No animal perceives the world as a sum of abstract and neutral aggregations in an otherwise dumb Euclidian space. They contextualize the world into sense-making environments – an obvious precondition for orientation, survival and reproduction, which lead Gibson (1979; Gibson 1988) to reject the conception of ‘physical environment’ for ecological thinking. The organism is the precondition for something to be an environment after all. This was already acknowledged by Jakob von Uexküll, who introduced the term Umwelt, defined as the surroundings as experienced by the subject (Uexküll and Kriszat 1970, p. 107 ff). While the ecological niche sums up the factors and objects which affect fitness of a given species as seen from the outside, Umwelt is the subjective and sense-making version of the same, focusing on what the ecological niche means when seen from the inside. The sum of signs perceived by the subject defines its semiotic niche (Hoffmeyer 2008; Kull et al. 2009; Packard and Delafield-Butt 2014). Uexküll circumscribed it as a musician relation, where the intercept for adaptive change expresses the emerging melody of the tuning of organisms to the tunes from the Umwelt.

And subjects learn - they gain new information or skills, and change behavior accordingly (Pearce 2008; Sznajder et al. 2012), physiologically enshrined as new neuronal connections in response to environmental stimuli (Koizumi 2004). Learning comprises the flexible intercept between cultural influences, Umwelt and neurobiology. Mechanical systems do not learn. They repeat errors and collapse when the malfunctioning part breaks, because they lack an inherent power to adjust behavior (Fitch 2005). Things and computer programs perform what Ciliberti et al. (2007) call “brittleness”, where modification of one component easily lead to disastrous failure of the entire system. Living systems, on the other hand, comprise “anti-fragility” (Danchin et al. 2011b), pointing to resilient mechanisms for correcting errors on multiple levels: epigenetic, physiological and developmental, which sum up to phenotypic plasticity. This flexibility is a prerequisite for evolvability and speciation, and its behavioral counterpart is learning. The possible influence of learning for variation and lineage splitting has followed evolutionary thinking since the advent of Darwinism, as Henry F. Osborn, Conwy Lloyd Morgan and James M. Baldwin independently pointed to learning as a potential source for novelties, in what occasionally was termed “organic selection” (Baldwin 1896; Lloyd Morgan 1896; Osborn 1896; Hall 2001; Weber and Depew 2003). But its significance was left largely unnoted within the framework of the Modern Synthesis, as reflected in a low rate of published research on learning during the second half of the 20th century - followed by an explosive increase in publications during recent years, however.

Today learning is acknowledged as a common feature across the entire animal kingdom (Plotkin 1988; West-Eberhard 2003: 337–352; Bateson 2004; Galef and Laland 2005; Shapiro 2011; Lindholm 2012; Dukas 2013). Any species-specific morphology - whether beaks, fins, tails or legs - evokes particular behavioral patterns, which are reinforced and guided through development and adolescence by learning. This morphology-biased learning capacity (West-Eberhard 2003; Bertossa 2011) is the glue which connects development, physiology, epigenetics and behavior, and fine-tunes them to the environment. Galef and Laland (2005, p. 489) accordingly define ‘adults’ as individuals which have learned how to live: “Adults are individuals that have acquired patterns of behavior allowing them to avoid predators and the ingestion of toxins, to select an adequate diet, and to find water and safe refuge”.

Learning capacity clearly increases with neural complexity, but is observed in species with simple nervous systems, as well (Linsenmaier 1987; Krasne and Glanzman 1995; Libersat and Gal 2013; Vitti 2013). The upcoming science of behavioral epigenetics has from various angles explored how learning and memory shape the epigenome of the brain, and experimental studies have revealed that learning associates with chromatin remodeling, in terms of phosphorylation, acetylation and methylation in neural networks (Levenson and Sweatt 2005; Day and Sweatt 2010; Puckett and Lubin 2011; Renn and Schumer 2013; Snell-Rood 2013). Moreover, exercises and repetition establish new synaptic circuits, which interconnect and consolidate learned skills, leading Jean-Pierre Changeux to characterize learning as the art of “stabilizing pre-established synaptic combinations” (Changeux 1985, p. 248).

In vertebrates the neural formatting for behavioral guidelines is fully integrated in early development. Weaver et al. (2004) revealed that pup licking and grooming in rodent mothers caused increased densities of glucocorticoid receptors in the CNS of offspring, which was associated with enhanced tolerance against stress. Yet, this learned behavior has a heritable component, as pups exposed to grooming later tend to behave accordingly to their own progeny. Similarly, prenatal auditory song perceptions are prerequisites for later song learning and recognition of conspecifics in many birds (Gottlieb 1971).

West-Eberhard (2003) describes learning as a condition-sensitive decision process, because behavioral change due to learning requires decisions. The problem of how decision making per se is possible has largely escaped the discussion, despite efforts to analyze its premises (Pelé and Sueur 2013). Mechanistic systems neither learn nor decide, but perform behavioral patterns which may be fully explained by their determinants. Learning relates to a subject who recognizes the error in time and decides to adjust behavior accordingly, even across physiological determinants and genetic guidelines. Preconditions cannot decide, however. Decisions need a decider, a proactive subject which considers, foresees and judges (Plotkin 1988; Bateson 2004; Fawcett et al. 2013). This was conceded within the frame of the Modern Synthesis, as well, by claims such as “we can indeed override our genes” (Dawkins 1976, 2003), but without further elaboration.

Subjects foresee, decide and respond purposely due to inherent motives and “beliefs”, which partly are created by themselves and not by external factors (Sterelny 2003; Fitch 2008; Northoff and Panksepp 2008). As such, subjects are not merely present endpoints of the past, but also potential starting points for novelties. Animal evolution is therefore not only a matter of re-action, but also of action. Through learning individuals begin to decide over themselves, and decision making depends on autonomous systems which cannot be reduced to an effect of genes (Nagel 1974; McGinn 1999; Fitch 2008; Rosslenbroich 2009, 2014). This disposition for behavioral autonomy has a physiological basis in the basal ganglia system (Stephenson-Jones et al. 2011), which is deeply rooted in vertebrate phylogeny. Even ancestral jawless fish such as lampreys comprise similar dual behavioral motor output systems like those of mammals, and their basal ganglia are fully developed. Lampreys are hence capable of regulating behavior by both direct and indirect motor output pathways from the basal ganglia, and possess the neurological basis for decision making (Stephenson-Jones et al. 2012; Mashour and Alrike 2013).

Further support for the potential integrity of subjects is the widespread pattern of maladaptive behavioral syndromes, where individuals maintain sub-optimal behavior despite significant costs (Bednekoff and Lima 1998; Sih et al. 2004; Adriaenssens and Johnsson 2013). Examples include high activity levels despite presence of predators, as in African springboks (*Antidorcas marsupialis*), in newts opposing predatory fish, lemming in northern alpine habitats, or exaggerated sexual cannibalism in certain spider species (Maupin and Riechert 2001). Similarly, learning has repeatedly been observed to generate misconceptions that make subjects prone to fatal mistakes and accidents, especially among young and inexperienced individuals (Wuczynski and Jakobiec 2013).

How learning abilities have contributed to novelties and speciation has been thoroughly explored from various angles by researchers working with a taxon repeatedly referred to by the Modern Synthesis, namely Darwin’s finches on the Galapagos archipelago. A closer look into this case may reveal how learning has affected three core areas of natural selection, *food choice*, *mate choice* and *habitat choice*, and thereby contributed to lineage splitting.

## Speciation in Darwin’s Finches

The Galapagos archipelago has a long history in biological thinking (Darwin 1845; Wallace 1855). Already in his first article on evolution Alfred Russel Wallace claimed that the original colonists on the islands must have descended from the eastern continent, and that their isolated position had led to new species:

We can account for the separate islands having each their peculiar species, either on the supposition that the same original emigration peopled the whole of the islands with the same species from which differently modified prototypes were created, or that the islands were successively peopled from each other, but that new species have been created in each on the plan of the pre–existing ones. (Wallace 1855, p. 6)

Wallace had a particular passion for birds, and had certainly the various species of finches (*Geospizinae*) in mind, today named after Darwin, who sampled them during his journey with Beagle, but without noticing their puzzling local distribution. The differences between the fourteen species are primarily associated with beak shape and song patterns. Molecular studies point to a flock of finches that presumably arrived on the Galapagos from the mainland some two million years ago (Burns et al. 2002). They faced an alien and heterogeneous nature of mangrove woods, humid forests, montane steppes and rocky deserts. The new world comprised few competitors and predators, and geographic isolation in combination with genetic drift and random mutations facilitated local genotypes, which caused the founding population to radiate into a whole venue of closely related species. Foraging seems to have been a central driver for local adaptations. Hard seeds favored broad and deep beaks (as in ground finches, *Geospiza fuliginosa*, *G. fortis* and *G. magnirostris*). Other specialized on fruits, pollen and cactus flowers and developed long pointed beaks (cactus finches, as *G. scandens* and *G. conirostris*). Some turned to insectivory, and at least one species - the woodpecker finch (*Camarhynchus pallidus*) - even masters the art of poking insect larvae from crevices in trees by use of sticks and spines.

### Learn What to Feed on

The story of Darwin’s finches neatly matches the core concept of allopatric speciation as predicted in the Modern Synthesis: Random changes in allelic frequencies allow fitness to vary, which in combination with geographic isolation - the proximity of the various islands was small enough to allow episodic events of migration, but mainly large enough to ensure reproductive isolation - eventually lead to new species.

A broader view of the Galapagos fauna elicits new questions, however. The total numbers of birds on the islands amounts to 30 (seabirds not included), and of these are 14 finches. Among the others only one taxon shows any sign of radiation (four new species of mockingbirds, *Nesomimus sp.*). The remaining twelve species have not undergone any speciation at all. A similar pattern is observed in other taxa. Rodents show some degree of speciation, while bats do not. Reptiles have split in 4 of 7 cases. Among the 1500 insect species on Galapagos there are hardly any new species (Parent et al. 2008). Thus, the impressive radiation in Darwin’s finches is clearly not part of a general pattern. All colonists found similar unoccupied niches at the archipelago, but their evolutionary response differed.

Essential for survival in foreign environments is diet and food choice. In mammals initial dietary preferences are already established in utero, as the fetus gets familiar with the mothers diet through the placenta (Galef and Laland 2005). Pups gain further dietary information through scents from mothers fur, mouth and urine, and through lactating, where new flavors from her diet are mediated (Beauchamp and Mennella 2009). Most birds feed on a vast array of food items, dependent on habitat features and seasonal opportunities, where memory and learning are central prerequisites (Smulders and Dhondt 1997; Bairlein 2002; Tilgar et al. 2002; Slagsvold and Wibe 2007). Nestlings inherit food preferences from their parents, which mediate local traditions based on their own infancy (Avital and Jablonka 2000). Food choice is also affected by the amount of explorative willingness (Brown 2013; Carere and Maestripieri 2013; Dingemanse and Wolf 2013). Dietary innovative taxa are observed to have higher speciation rates (Nicolakakis et al. 2003), probably due to their greater ability to explore new niches and habitats (Sih et al. 2004). An example is the blue tits in the UK, which pierced through the foil cap of milk bottles left outside house doors, in order to drink the cream. This habit dispersed rapidly and was even absorbed by other species. Reconstructions later revealed that the innovation spread from local epicenters, probably by means of social learning (Fisher and Hinde 1949; Aplin et al. 2013). Such learning-driven behavioral shifts open new resources for the population, but expose them to modified selection pressures, too. Mutations which facilitate the behavior will be favored, at the expense of individuals who need to learn from scratch. Skills that were originally purely behavioral may hence be gradually assimilated by genes (Waddington 1961; West-Eberhard 2003; Pigliucci et al. 2006; Badyaev 2009; Renn and Schumer 2013). Selection on other traits would also be likely, in the latter case for instance the ability to digest chemical components of milk.

Evidence for such subsequent adaptations to behavioral driven food choice is well known in the fossil record. A high-resolution record of Miocene sticklebacks (*Gasterosteus sp.*) revealed a change in dental microwear, reflecting a dietary shift from pelagic zooplankton to a benthic diet, associated with a change from pelagic to benthic habitat. The shift was accomplished by a corresponding enlargement of spines and body armour, assumingly associated with increased predation. The dietary shift, however, preceded increased body armour by 200 years (Purnell et al. 2007; Lister 2014). Sticklebacks first changed diet and habitat, and later morphology followed. Similarly, increase in African herbivore proboscideans molar crown height (hypsodonty) - a morphological adaptation to a more abrasive diet dominated by grasses - significantly lagged after the species changed habitat from woodland to open savannah grassland during the Tertiary (Neogene) period (Lister 2013, 2014).

Learned food choice has certainly contributed to speciation in Darwin’s finches, as well (Grant 1986). West-Eberhard (2003) and Grant and Grant (2008) reviewed the unusual flexible feeding techniques in their foraging habits, and comment on their striking explorative willingness. Several of Darwin’s finches use tools, similar to the woodpecker finch, to pick out caterpillars and insects from crevices in bark or cracks in stems. The vampire finch (*Geospiza difficilis septentrionalis*) pecks the skin of other bird species and drinks on the blood droplets, or pushes gannet eggs out of nests and feeds on the smashed remains. Others strip bark from trees or break up cactus buds and feed on pollen. Some Darwin’s finches look for bugs by kicking over stones, pick parasites from the skin of lizards or catch spiders by pulling them out by their silk thread with their feet. To quote a recent study:

We must imagine that flexibility allowed finches to respond to environmental variation by adapting differently to different habitats and to the resources available within them. These differences would initially have been merely behavioral, upheld by common mechanisms of learning, but later genetic accommodation would have entrenched some behavioral differences and supplemented them with morphological differences, as morphology co-evolved with behavior. In this model, differences that are dependent on learning appear first, to be followed by genetic and morphological differentiation and ultimately by isolation and speciation. (Tebbich et al. 2010, p 1101).

Fitness differences in Darwin”s finches have initially probably been driven by behavioral flexibility and explorative willingness. Founder populations are known to be particular vulnerable to extinction due to lack of adaptations to the new environment (Bouzat 2010), as mutations or genetic drift respond too slowly to mitigate the new demands. Under such circumstances learning abilities are especially important, as they may bridge maladaptive physiological or morphological limitations (Kokko and Sutherland 2001; Vander Wal et al. 2012), and allow the genome time to adapt.

### Learn who to Mate

As with food choice mate choice is a matter of behavioral flexibility in a wide range of animal taxa (Avital and Jablonka 2000; Freeberg 2000; White 2004; Hebets and Sullivan-Beckers 2010; Verzijden et al. 2012), including polychaetes (Fletcher et al. 2009), crustaceans (Shuster and Wade 1991), cephalopods (Huffard et al. 2008), insects (Bonduriansky 2001; Mery et al. 2009; Westerman et al. 2012; Dukas 2013) and vertebrates (Dugatkin and Godin 1992; Penn and Potts 1998; ten Cate and Vos 1999; Galef et al. 2008). Male Trinidad guppies (*Poecilia reticulata*) reared in isolation even fail to recognize females of their own species (Witte and Nöbel 2011), and experimental cross-fostering has repeatedly induced lifelong sexual preferences for foster species mates (Kendrick et al. 1998; Slagsvold and Wiebe 2007; Hebets and Sullivan-Beckers 2010), suggesting that even fundamental faculties of fitness depend on learning.

The importance of learned vocal courtship in birds has been extensively studied, especially in passerine songbirds. Song is learned through a succession of stages which may cover the entire life history. Initial imprinting begins during embryonal development, followed by the nestling listening to adult song, later attempts at song reproduction, which by means of exercise and improvisation finally lead to the adult song repertoire. The learning history involves a finely tuned interplay between developing brain structures, neuro-chemical systems, epigenetics, learning and error detection. European starlings (*Sturnus vulgaris*) continue to add new song motifs from year to year, frequently integrating human sounds such as music, engines or hammering, with length and variation of song bouts differing both between and within cohorts (Gentner and Hulse 2000). Migratory bluethroats (*Luscinia svecica*) arriving higher latitudes from Africa in spring frequently include calls learned from their tropical counterparts. Song repertoire interacts with the size of the neural song-control nuclei, which develops during adolescence and is affected by diet quality and learning abilities, hence reflecting cognitive capacity, which also serves as a fitness marker for sexual selection (Darwin 1871; Nowicki and Searchy 2004; Boogert et al. 2011).

In sympatric Darwin’s finches where reproductive isolation is partly incomplete and learned song differences serve as pre-mating barriers, hybridization has repeatedly occurred as a result of misinterpreted male songs. Both female hybrids and their offspring prefer males which sing the songs of their fathers (Grant and Grant 1997). This was elaborated by Grant and Grant (2009), who observed an immigrant medium ground finch (*Geospiza fortis*) male on Daphne Major Island, which deviated from the main population through a slightly different beak morphology and peculiar song pattern. It gave rise to a new lineage, with females solely reproducing with males of their own lineage, suggesting the departure of a new species, based on learning induced variation. Learning was assumed to contribute to isolation both through morphology and song pattern in this case, because beak shape serves as a marker for conspecifics and is transferred to offspring through learning (Grant and Grant 1997), similarly to male song pattern.

### Learn Where to Live

The ability of organisms to identify particular and suitable habitats and stick to them across generations is a basic premise for natural selection – adaptive advantages would otherwise be as changeable as the weather. How this faithfulness is maintained has puzzled scientists for a century (Elton 1927, p 39–42; Waddington 1959; Hardy 1965; Hinton and Nowlan 1987; Rodenhouse et al. 1997; Bateson 2004), but was left astonishingly uncommented during the era of the Modern Synthesis (Jones 2001; Piper 2010). Some textbooks admitted that “Habitat selection is one of the most poorly understood ecological processes” (Krebs 1994, p. 61), but most left the topic completely unmentioned (Begon et al. 1990; Ridley 1993; Campbell 1996). Even textbooks in biogeography avoided the puzzle, or wrote in passing vaguely about “behavioral or psychological barriers” (Brown and Lomolino 1998, p. 165).

An increasing body of evidence has proven that the ability to identify suitable habitats according to specific environmental features is a common feature of animals, and learning of habitat cues has been demonstrated for a large number of taxa, from mollusks and arthropods to fish, birds and mammals (Scapini 1988; Haas 1998; Svensson et al. 2000; Hoover 2003; Davis 2008; Mabry and Stamps 2008; Stamps et al. 2009).

Some preferences are individually learned early in life, as when anadromous fish return to their natal river or when ovipositioning insects return to the site where they hatched (Refsnider and Janzen 2010). In birds habitat choice is crucial during nesting and reproduction, and birds easily distinguish between low- and high-quality habitats, and size up the territory accordingly (Fretwell and Lucas 1970). Doligez et al. (2002) demonstrated how learning determines habitat choice in collared flycatchers (*Ficedula albicollis*), by artificially distorting part of the brood from nests in one area and adding them to nests in another. The latter area hence appeared more suitable for reproduction for visiting conspecifics, and this area was inhabited by significantly more flycatchers the following year. Seppänen and Forsman (2007) demonstrated that habitat choice of one species also affects decision making in other. After having brought out birdhouses for tits in their research area, they marked the front of occupied birdhouses with white circles, and vacant ones with triangles. As migratory flycatchers later arrived, they hung up additional birdhouses, now equipped with both triangles and circles. The flycatchers, however, exclusively settled in birdhouses with circles. They seemingly had observed the brood differences of the two birdhouse types, and preferred the houses which appeared to be more successful.

Habitat preferences may be affected by genetic inheritance (Jaenike and Holt 1991; Via and Hawthorne 2002), but learning has repeatedly trigged habitat shifts, which has led to genetic change and eventually speciation, similarly to the Miocene sticklebacks and African proboscideans. A more recent example is the ongoing urbanization of European blackbirds (*Turdus merula*). The species was originally limited to forests, but some individuals invaded the city of Erlangen (Germany) in 1820. Although the urban habitat profoundly differed, they managed to breed and interacted successfully with the foreign competitors, predators and diseases (Evans et al. 2010). The growing urban population gradually spread, and is now present in most cities of Europe. Urban blackbirds rarely interbreed with the rural, and differ both genetically and behaviorally from their ancestors (Mueller et al. 2013). If the new habitat remains stable the lineage split will possibly deepen, and a new species could form.

The conventional premise has been that natural selection is blind and without mind or purpose: “The process cannot have a goal, any more than erosion has the goal of forming canyons, for *the future cannot cause material events in the present.* Thus the concepts of goals or purposes have no place in biology” (Futuyma 2013). This is, however, not quite true. Not all animal species purposely create new structures, as through niche construction, and they have no evolutionary agenda, indeed, but they take numerous actions in order to choose habitats which promise a better match with their own needs. In fact, to some extent animals actively choose which selection regime to which they will be subjected (Elton 1927; Bateson 2004).

In clades of patchy distributed and closely related species, as in Darwin’s finches, the learning of habitat features is a significant contributor to speciation (Beltman and Metz 2005). Grant et al. (2001) monitored during three decades the development of a new founding population of the large ground finch (*G. magnirostris*) on Daphne Major Island. Stray birds had been visiting this small island irregularly since 1973 during the dry (non-breeding) season. In 1982, however, migrants arrived even during the wet season and a first brood appeared, which became founders for a growing population. Ever new stray birds visited the island for shorter or longer periods during the following years, and some bred. Two song patterns inherited from the founders were maintained for the following period, before a new song gained dominance. Molecular microsatellite analyses revealed that the immigrants came from four different islands. However, the settlers were not a random subset from the source areas, as most came from one particular island. The immigrants seemingly scanned the novel habitats for certain preferred features wherever they arrived, and thereafter chose to breed or to move on (Tonnis et al. 2005). The emerging ‘cultural species’ (Beltman et al. 2004) became rapidly exposed to natural selection. During a severe drought in 1982 all individuals with pointed beaks starved to death. During the following years, the population had a more blunted beak shape, demonstrating the rapid transformation of a ‘cultural’ into a genetic speciation (Grant et al. 2001).

## Learning and the Extended Synthesis

A closer look at Darwin’s finches reveals that cognitive abilities and learning clearly have been necessities for successful speciation even in this canonical story of the Modern Synthesis. Learning, and decisions are potent sources for variation and novelties, and the process of speciation is not solely determined by the population history, as enshrined in the genes and driven by random change in allelic frequencies. What subjects purposely decide to do on the basis of experience and learned skills contribute, as well. Organisms are hence dynamic arenas of the deterministic past and the innovative future, and the science of biology accordingly explores the interfaces between determinism and freedom. If life was merely a matter of the deterministic past, nothing would remain to wonder about. And was it solely unpredictable creativity, nothing would be left to reason about and analyze. Genes mediate the echo of the evolutionary history into the present. But the future is also present, through the attempts of the imperfect, but innovative subject. Speciation can hence be defined as the instantaneous interface between the past and the future. A similar perspective was emphasized by Odling-Smee et.al. (2003, p. 179), in characterizing niche construction:

natural selection is goalless [… and] is an uninformed or blind selective process. By “blind” we mean that natural selection, unlike niche construction, is not guided by any kind of registration, or memory, of the past. Nor, unlike artificial selection, is it prescient. It cannot prepare for or predict future organism-environment interactions “here and now”. Niche construction is pro-active (…). Natural selection is reactive.

Learning may clearly be genetically influenced as an integrated part of the developmental plasticity of the phenotype, and as such a matter of natural selection. But preconditions can only facilitate, not generate, learning and decisions. Learning and decisions concern the first-person perspective (Rudder Baker 1998; McGinn 1999; Davis 2004), making sense only from an autonomic and subjective “inside”, and are from outside solely deducible through observation of behavioral change, or as electric signals along neural pathways. Both physiology and genes facilitate behavioral flexibility, but learning itself can solely be anticipated from the “inside”. Learning-based decisions are not features, but events, ruled by the motive-driven subject, and not by DNA. Genes may dispose, but they cannot decide. The learning event is the moment where the subject faces its world with fresh eyes, and such moments comprise the potential to change behavior and thereby fitness.

The fact that learning is independent of genes was in reality also acknowledged by Richard Dawkins (1976), reflected in what he called memetic inheritance (Marsden 1998; Aunger 2001). How well the meme concept explains the impact of culture on evolutionary novelties remains open (Rose 1998; Kull 2000; Deacon 2004; Distin 2005). But it underpins the fact that even proponents of the Modern Synthesis recognized the necessity of sources of biological novelties independent of genes. Memes are thought of as cultural elements that subjects partly inherit and partly create. But more importantly, they may invoke heritable variation, analog to genes: “Memes and genes may (…) sometimes come into opposition. For example, the habit of celibacy is doomed to failure in the gene pool (…). But still, a meme for celibacy can be successful in the meme pool*”* (Dawkins 1976). Ideas of how memes contribute to fitness shifts also appeared in popular science. Memes “changed the environment in which the genes were selected, and so forced them to build better and better meme-spreading apparatus. (…) Successful memes begin to dictate which genes are most successful” (Blackmore 1999, p. 99). The cultural factors here circumscribed as memes is considered as a separate, gene-independent source of heritable variation, which also may guide genes - “genes as followers” (Pigliucci and Müller 2010).

If left alone, genes must appear to be almighty evolutionary determinants, which could even be the ultimate explanation for the Modern Synthesis itself, simply due to the lack of any other possible source for novelties. But such a scientific fundamentalism inevitably generates absurd self-reference-problems (Fitch 1946, 2008; Rorty 1979; Hughes 1982), making it impossible to test hypotheses, as any objection could be viewed as part of the theory claimed. Human learning link scientific theories to cultural arenas independent of genes, and clarifies what is inside and what is outside the scientific models. The gene independent act of learning and decision making hence represents a substantial extension of evolutionary thought, which also assures the Modern Synthesis as a scientific theory.
